# Quantitation of isobaric phosphatidylcholine species in human plasma using a hybrid quadrupole linear ion-trap mass spectrometer[S]

**DOI:** 10.1194/jlr.D070656

**Published:** 2016-11-28

**Authors:** Petr Zacek, Michael Bukowski, Thad A. Rosenberger, Matthew Picklo

**Affiliations:** USDA-ARS Grand Forks Human Nutrition Research Center,* Grand Forks, ND 58203; Institute of Organic Chemistry and Biochemistry Academy of Sciences of the Czech Republic,† 16610 Prague 6, Czech Republic; Department of Biomedical Sciences, University of North Dakota School of Medicine and Health Sciences,§ University of North Dakota; Grand Forks, ND 58201; Department of Chemistry,** University of North Dakota; Grand Forks, ND 58201

**Keywords:** triple quadrupole/ion-trap, National Institute of Standards and Technology human blood plasma, mass spectrometry, shotgun lipidomics, docosahexaenoic acid

## Abstract

Phosphatidylcholine (PC) species in human plasma are used as biomarkers of disease. PC biomarkers are often limited by the inability to separate isobaric PCs. In this work, we developed a targeted shotgun approach for analysis of isobaric and isomeric PCs. This approach is comprised of two MS methods: a precursor ion scanning (PIS) of mass *m/z* 184 in positive mode (PIS *m/z* +184) and MS^3^ fragmentation in negative mode, both performed on the same instrument, a hybrid triple quadrupole ion-trap mass spectrometer. The MS^3^ experiment identified the FA composition and the relative abundance of isobaric and *sn-*1, *sn-*2 positional isomeric PC species, which were subsequently combined with absolute quantitative data obtained by PIS *m/z* +184 scan. This approach was applied to the analysis of a National Institute of Standards and Technology human blood plasma standard reference material (SRM 1950). We quantified more than 70 PCs and confirmed that a majority are present in isobaric and isomeric mixtures. The FA content determined by this method was comparable to that obtained using GC with flame ionization detection, supporting the quantitative nature of this MS method. This methodology will provide more in-depth biomarker information for clinical and mechanistic studies.

Phosphatidylcholines (PCs) are the most abundant phospholipid species in human plasma (1, 2). Changes in the PC species of various tissues are linked to cardiovascular disease, diabetes, and cancer (3–7). A classical method for the analysis of PCs is HPLC in connection with MS detection with ESI (2, 7, 8). Progress in MS instrumentation has enabled the replacement of the separation step by direct injection of a sample into the mass spectrometer, also known as shotgun lipidomics (9, 10). Removing the chromatography step considerably reduces the total time of analysis. However, a shotgun approach requires special attention to sample concentration, mobile phase (MP) composition, and ionization mode selection to minimize matrix effects for quantitative purposes (9–16).

Clinical lipidomic studies suffer from a lack of structural information of the analyzed PCs (17–19). In many studies, PCs are identified only as the number of carbons and double bonds. While this information is useful, identification only to this level of structure is problematic. Missing constitutional and positional information may lead to the misinterpretation of the role of PCs in selected biological processes (19–23). For example, decreases in plasma levels of PC 38:6 are linked to progression of mild cognitive impairment to Alzheimer’s disease (19). However, PC 38:6 may consist of at least six isobaric and isomeric species, including PC 16:0/22:6, PC 22:6/16:0; PC 18:1/20:5, PC 20:5/18:1; PC 18:2/20:4, and PC 20:4/18:2. The classical analytical techniques for the determination of constitutional and positional isomers involve a combination of laborious and time consuming chromatography, chemical transformations such as transesterification, and enzymatic reactions with lipases, particularly phospholipase A_2_ (PLA_2_), for the determination of positional isomers (15, 24). Differential ion mobility spectrometry is a promising technique for the differentiation of PC positional isomers after adduction with silver ion (25). However, this technology is currently limited to a rather low number of MS platforms.

PCs can be analyzed either in positive or negative mode (26–30). In this study MS^n^ fragmentation in negative ESI mode is employed to quantify isobaric and isomeric PCs (28, 29). In the negative mode, MS^n^ fragmentation of PC counter-ion adducts (chloride or acetate) results in the loss of the anion and elimination of a methyl group from the quaternary nitrogen of the choline head group [PC-15]^−^. MS^3^ fragmentation of the [PC-15]^−^ ion produces a demethylated lyso-PC (dLPC), [dLPC]^−^, and a FA anion [FA]^−^ (28). The ratio of peak heights for [dLPC]^−^ having a FA in the *sn-*1 position [*sn-*1_dLPC]^−^ is proportional to the abundance of the isomeric PCs. Based on these observations, it was suggested that these fragments could be used for identifying the positional isomer composition of isomeric PC species (28, 31). A resulting analytical method that was suggested required two instruments working in tandem: *1*) a quadrupole-time-of-flight instrument to perform a positive ESI precursor ion scanning (PIS) for the choline head group (*m/z* 184) and a negative ESI PIS for FA composition; and *2*) an ion trap instrument to perform the negative ESI MS^3^ scans needed for the determination of the positional isomers (10, 13, 28).

Based upon these prior observations, we developed a quantitative shotgun approach for the analysis of isobaric and isomeric PC species. Our method is comprised of the direct injection of a crude organic plasma extract followed by analysis with a combination of both PIS of *m/z* 184 in positive mode (PIS *m/z* +184) and MS^3^ fragmentation methods, performed on the same instrument, a hybrid quadrupole with a linear ion-trap mass spectrometer. We compared the use of resulting [dLPC]^−^ and [FA]^−^ fragments after MS^3^ for isobaric and isomeric PC quantitation and validated this approach using a NIST human blood plasma standard reference material (SRM) (SRM 1950).

## MATERIALS AND METHODS

### Chemicals

The following chemicals were purchased from Sigma-Aldrich (St. Louis, MO): chloroform, potassium chloride, butylated hydroxytoluene (BHT), ammonium chloride, ammonium acetate, and PLA_2_ isolated from porcine pancreas. Methanol was purchased from Avantor Performance Materials, Inc. (Center Valley, PA); 18 MΩ water was used. A solid unmodified silicic acid 200–325 mesh from Clarkson Chromatography Products Inc. (South Williamsport, PA) was used as a stationary phase for solid phase extraction (SPE). Nonadecanoic acid and FA methyl ester calibrants for plasma FA analysis using GC coupled with flame ionization detection (GC-FID) were purchased from Nu-Chek Prep, Inc. (Elysian, MN).

All phospholipid standards used in this study, including LIPID MAPS MS standards-Core H, were purchased from Avanti Polar Lipids (Alabaster, AL). Human plasma SRM (SRM 1950) was purchased from the National Institute of Standards and Technology (NIST; Gaithersburg, MD). This material was prepared by collecting plasma samples from healthy people 40–50 years of age and represents the ethnic distribution of the US population.

### Plasma sample preparation

An organic extract of the plasma was prepared based upon the procedure of Folch (32, 33). Plasma (100 μl) was placed into a 5 ml glass test tube and 2.5 ml of extraction solution chloroform:methanol (2:1, 50 μM of BHT) and 0.5 ml of water solution of potassium chloride (0.9%) were added. This mixture was vortexed (1 min) and then centrifuged (10 min, 3,000 *g*, 15°C). The lower phase was removed and placed into a 15 ml glass test tube. The process was repeated on the remaining aqueous phase by adding 2.5 ml of chloroform with BHT. The organic phases were pooled and the solvent evaporated under a N_2_ stream at 30°C. The dried extract was reconstituted in 1 ml of chloroform to yield the crude extract.

The extraction efficiency of the above introduced procedure was tested by measuring the abundance of PC 13:0/13:0 and PC 21:0/21:0 between two samples. In the first sample, the PCs were added directly to the plasma prior to the extraction, while in the second sample, the two PCs were added to the final plasma extract. Comparison of PIS *m/z* +184 (see below) signals of the PCs between both samples showed the extraction efficiency to be higher than 90% for both PCs.

### SPE separation

Plasma lipids were separated using silicic acid SPE. Glass columns (0.85 cm internal diameter; Macherey-Nagel, Dürer, Germany) were filled to a 1 cm height of stationary phase consisting of a solid unmodified silicic acid 200–325 mesh. The stationary phase was preconditioned in chloroform (50 g of solid unmodified silicic acid in 1 l of chloroform) for at least 24 h prior to use. Each column was equilibrated with 2 ml of chloroform before sample loading. All of the plasma chloroform extract was loaded on the SPE column. The neutral lipid (NL) fraction was eluted first using 5 ml of chloroform containing BHT (50 μM) followed by 2× 3 ml of chloroform:methanol (58:1, with 50 μM BHT). Polar lipids (PLs) were eluted using 2× 3 ml of a mixture of methanol:water (95:5, 50 μM BHT). The PL fraction was dried down under N_2_ stream at 30°C and reconstituted in 1 ml of MP consisting of chloroform:methanol (1:1, with 10 mM ammonium acetate) for MS measurements.

### Hydrolysis by PLA_2_

PLA_2_ was used for determination of the isomeric purity of synthetic standards, PC 16:0/18:1 and PC 18:1/16:0. The enzymatic reaction followed by sample preparation for MS analysis was carried out as described in Ekroos et al. (28).

### TLC analysis

A portion (0.95 ml) of crude plasma organic extract was dried under N_2_ and reconstituted in 0.1 ml of chloroform. A portion (75 μl) was loaded on a TLC plate (Whatman Silica Gel 60A LK6, 20 cm^2^) and separated using a MP consisting of CHCl_3_:methanol:acetic acid:water (50:37.5:3:2) (34). Phospholipid spots were visualized using iodine vapor and identified by comparison with retention behavior of PL standards analyzed on the same plate.

### MS analysis

MS analyses were performed on an AB Sciex 5500 QTRAP hybrid quadrupole ion-trap mass spectrometer with a Turbo Spray source (AB Sciex, Framingham, MA) operating at unit mass resolution.

Samples were analyzed using two methods. PCs, LPCs, and SMs were identified and quantified as a sum of isobaric species using PIS of *m/z* 184 in positive mode. Other parameters were set as follows: ion spray voltage, 5,200 V; source temperature, 200°C; curtain gas, 20 l/min; ion source gas, 15 l/min; declustering potential, 80 V; entrance potential, 10 V; collision energy, 35 V; scanning rate, 1,000 Da/s. The infusion rate was 10 μl/min.

The quantitative ratio of isobaric species and positional isomers were determined by MS^3^ scan in negative mode using the following parameters: ion spray voltage, −4,500 V; source temperature, 200°C; curtain gas flow, 15 l/min; ion source gas, 15 l/min; declustering potential, −100 V; entrance potential, −10 V; collision energy, −32 V; excitation amplitude, 0.180 V; excitation time, 25 ms; scanning rate, 10;000 Da/s. A unit resolution was applied. Dynamic filling time of the linear trap was used. The first precursor corresponded to mass of [PC+acetate]^−^ and the second precursor corresponded to [PC-15]^−^. Samples were infused at a rate of 10 μl/min. The MS^3^ targets were selected for PCs with abundance greater than 1% of the base peak height in PIS of *m/z* +184. In the case of the NIST human plasma sample, 22 PCs were selected for the MS^3^ experiment. MS^3^ fragmentation was applied automatically on every selected PC in the preprogrammed sequence of individual experiments. The MS^3^ scan duration for PC species ranged from 10 to 40 s, according to the abundance of the PC and number of isobaric and isomeric species. The longest scanning time of 40 s was applied on low abundant PCs containing more than two isobars. Sample analysis was approximately 10 min in duration for all MS^3^ experiments and 2 min for PIS *m/z* +184.

Data were collected using Analyst® version 1.6 software (AB Sciex) and processed in PeakView® version 2.0 (AB Sciex) and self-programmed Excel macros (Microsoft Office 2010, Microsoft). Quantitation of PIS *m/z* +184 analysis was carried out by LipidView^TM^ software. Several isotopic corrections were applied on both PIS *m/z* +184 and MS^3^ fragmentation analysis. The isotopic corrections of the PIS *m/z* +184 included correction for different isotopic distribution for analytes and standards and subtraction of the isotopic overlap from neighboring analytes. This correction was carried out automatically in the LipidView^TM^ software and was applied on all quantified compounds. Correction of the MS^3^ analysis was applied on selected species based on the level of their isotopic cross-contamination using an in-house developed calculator in Excel (Microsoft Office 2010, Microsoft) (see supplemental information, Isotopic correction of the MS^3^ scan).

For quantitation of PCs, LPCs, and SMs by MS the following procedure was used: A 5 μl portion of the reconstituted organic extract or PL fraction was diluted in 1,000 μl of MP. This concentration was used for isobaric and isomeric PC signal distribution using MS^3^ fragmentation in negative mode. For the analysis using PIS *m/z* +184, the solution was further diluted (50 μl was added to 500 μl of MP) and internal standards (ISs) were added (LPC 17:1, PC 12:0/13:0, PC 17:0/14:1, PC 17:0/20:4, PC 21:0/22:6, SM d18:1/17:0). For each PC and LPC, an IS was employed for the quantitation of lipid species in a particular mass range. Components of the plasma sample coeluting with IS were subtracted. Correction for the suppression of ionization efficiency, caused by the increasing number of carbons in the PC molecule, was applied within the mass range. The equation correcting the ionization efficiency was determined both for PCs and LPCs by measuring equimolar mixture of standards. In the case of LPCs, two standards were used, but for the NIST plasma sample analysis only LPC 17:1 was added to the solution.

PCs were quantified in two consecutive stages. Stage 1: for ESI PIS *m/z* +184, a peak height comprising the sum of isobaric PCs was compared with those of the closest ISs (see above). Stage 2: for negative mode MS^3^ analysis, targeted PCs were fragmented and the height of the signals corresponding to [FA]^−^, [FA-CO_2_]^−^, and [dLPC]^−^ was determined. The FA and FA-CO_2_, or dLPC anions were attributed to a particular PC and recalculated as signal distribution in unit percent. The peak height determined in stage 1 was distributed among the isobaric PCs determined in stage 2. The isomeric PCs were determined by a signal distribution between the dLPC ions of the same PC.

For quantitation of SMs, only one IS was used, SM d18:1/17:0. For the correction of decreasing ionization efficiency within the increasing molecular mass, we used a slope determined by use of PC standards (35). The correction equation was as follows: A_SM_ = IS_SM_ × [1/(−0.00226 × M_SM_ + 2.621)]; where A_SM_ is the corrected signal of the quantified SM, IS_SM_ is the signal of the IS (SM d18:1/17:0), and M_SM_ is the molecular mass of the quantified SM (see supplemental Fig. S1).

### GC-FID analysis

FA content in the PL fraction of NIST human plasma after SPE was determined. Prior to the GC-FID analysis, FAs in the PL sample were converted to methyl esters using a methanolic solution of acetyl chloride with nonadecanoate as an IS (36).

### Nomenclature

We used PL nomenclature according to Liebisch et al. (37). For example, 1-palmitoyl-2-oleoyl-*sn*-glycero-3-phosphatidylcholine is represented as PC 16:0/18:1; while PC 16:0_20:0 represents PC that contains palmitic and arachidic acid at undefined positions on the glycerol. For the purpose of this work, we refer to “isobaric PCs” as PCs with the same mass but containing different FAs. The term “isomeric PCs” refers to PCs having the same FAs, but different *sn-*1 versus *sn-*2 configuration (28).

## RESULTS

### MP for MS analysis

We studied the composition of salt additives to the MP that would enable a sensitive detection of PCs in both positive and negative ESI mode and that would be compatible with the sample preparation procedure. With the addition of 5 mM ammonium chloride, nearly all PCs were detected in negative mode as chloride adducts (data not shown) (28, 38); however, subsequent precipitation of ammonium chloride in the ion source generated considerable fluctuations in the ion current during spraying and signal instability (data not shown). We observed that substitution of ammonium acetate (10 mM) resulted in a more stable ion current and the generation of the PC-acetate adduct; therefore, 10 mM ammonium acetate was used in subsequent work (39). Due to the presence of endogenous chloride ion in plasma extracts PC-chloride adducts still remained, though only at about 14% the intensity of the corresponding PC-acetate signal (see supplemental Fig. S2).

### MS^3^ fragmentation of PCs in negative mode

**Fig. 1.**
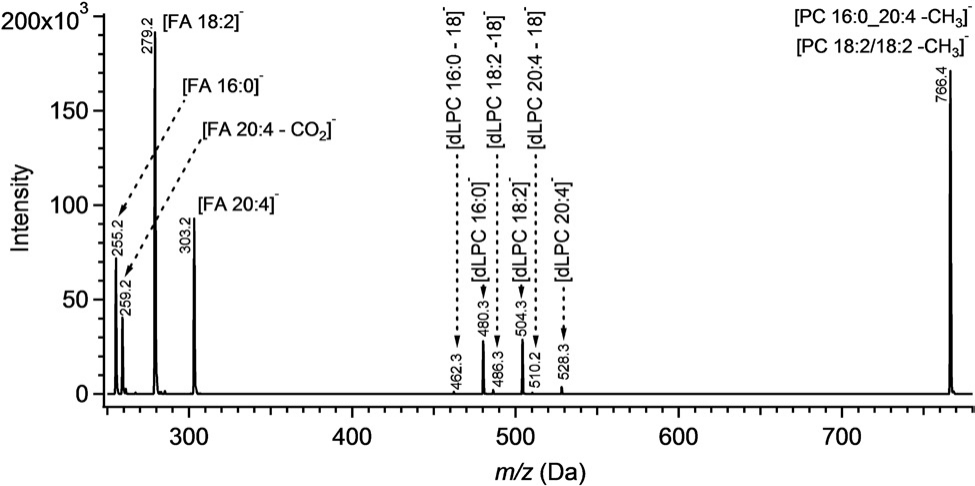
An MS^3^ spectrum of an equimolar mixture of isobaric PCs: PC1, PC 16:0_20:4; PC2, PC 18:2_18:2. dLPC fragments reflect the loss of the FA moiety as a ketene (28). An alternative fragmentation resulting in the loss of the neutral FA is represented as dLPC-18. PCs were analyzed as adducts with acetate in MP chloroform:methanol (1:1) with 10 mM ammonium acetate. Masses *m/z* 840.6 and 766.5 were selected for fragmentation.

In a tandem MS^n^ analysis in negative mode, fragmentation of PC acetate adducts generated ions corresponding to [PC-15]^−^, signifying the loss of the acetate counter ion and a methyl group from the quaternary amine of the choline moiety. Collision energy of 32 V maximized this signal, while higher collision energies increased the intensity of FA and FA-CO_2_ anions. MS^3^ fragmentation of anion [PC-15]^−^ resulted in a low relative abundance of the dLPC fragments with respect to the demethylated PC precursor and FA product ions (**Fig. 1**). While most FA species were detected as [FA]^−^, peaks corresponding to [FA-CO_2_]^−^ ions increased in intensity for polyunsaturated FAs proportional to the number of double bonds. The dynamic of the increase is more pronounced in the MS^3^ scan than in MS^2^ (see supplemental Fig. S3). Due to the observation that the intensity of the dLPC fragments is lower than FA fragments, we examined both product ion species for isobaric PC quantitation purposes in this work. Using FA anions for low abundance species analysis would be more sensitive in comparison to using dLPC anions.

### Isobaric PC composition determination

**Fig. 2.**
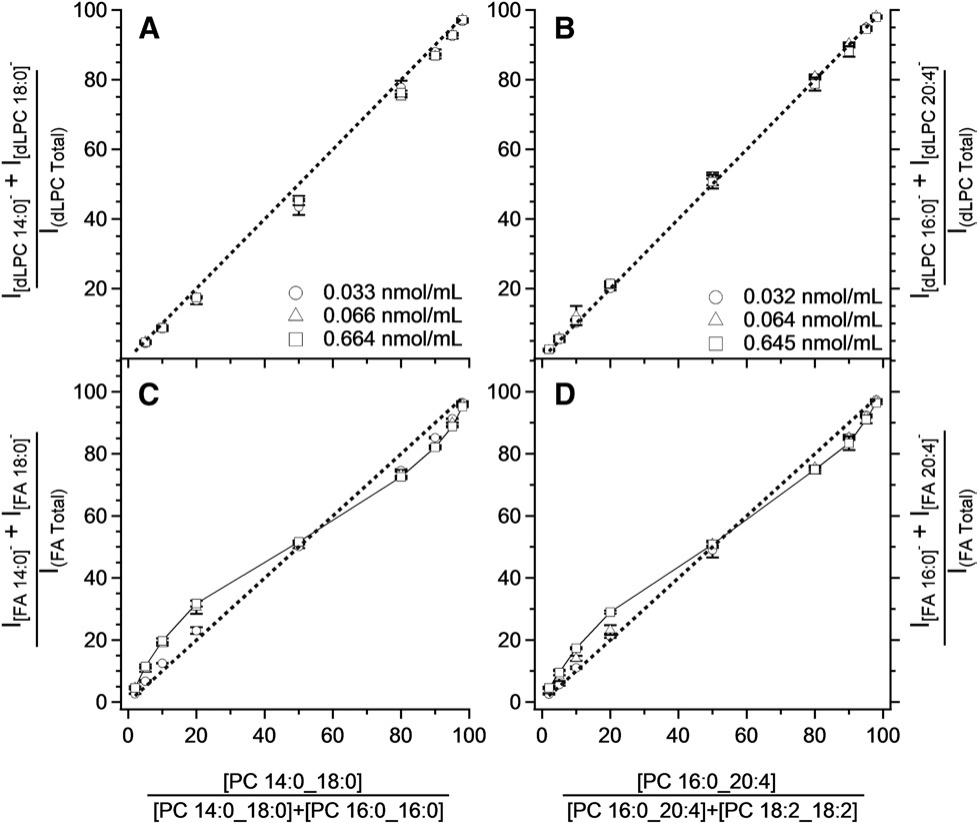
The correlation between estimated composition and composition determined using [dLPC]^−^ (A, B) and [FA]^−^ (C, D) ions after MS^3^ fragmentation of the isobaric PC mixture composed of saturated (A, C) and unsaturated (B, D) FAs. The concentration levels express the sum of the concentrations of both isobaric PCs in the particular saturated or unsaturated PC pair. The results for samples at 0.013 μM were identical to those observed for 0.033 nmol/ml and have been omitted for clarity. The dashed line shows where the points should lie in the case of an ideal agreement of the determined values with the actuals. Notice the sigmoidal dependency (C, D) when FA anions are used for composition of isobaric PCs at a concentration of 0.664 nmol/ml. The graph legends displayed in (A) and (B) are also valid for (C) and (D), respectively. The concentrations displayed in the legends exhibit the sum of the concentrations of examined pairs of PCs: PC 14:0_18:0 and PC 16:0_16:0 in (A) and (C) and PC 16:0_20:4 and PC 18:2_18:2 in (B) and (D). PCs were detected as acetate adducts. The analysis was performed using a MP consisting of chloroform:methanol (1:1) with addition of 10 mM of ammonium acetate. Bars represent mean ± SD of three repetitions.

Two sets of isobaric PC standards, PC 32:0 and PC 36:4, were used to validate the MS^3^ technique for determining constitutional isomers and to compare the suitability of using [PC-CH_3_]^−^ fragmentation products for such determinations. Samples composed of either PC 14:0_18:0 and PC 16:0/16:0 or PC 16:0_20:4 and PC 18:2/18:2 were prepared in nine different molar ratios and analyzed at four concentrations from 0.013 to 0.665 μM. The distribution of intensity of the product ions ([FA]^−^ + [FA-CO_2_]^−^) and [dLPC]^−^ of a particular PC are graphed against the molar ratios for the isobaric species in **Fig. 2**.

For both the fully saturated PC 32:0 system and the unsaturated 36:4 system, there is a strong correlation between the ratio of [dLPC]^−^ product ions and the molar ratio (see Fig. 2A, B). A line representing an idealized one-to-one correlation is displayed for reference, and is superimposable on the measured data for the desaturated PC species. While a second-order polynomial would present a closer fit for the saturated PC system, the more general linear model underestimates the amount of PC 14:0_18:0 in the isobaric mixture by about 5 mol% when relative composition of both examined standards (PC 16:0/16:0 and PC 14:0_18:0) is around 50%.

The correlation between [FA]^−^ fragments and molar ratio for isobaric species was sigmoidal and concentration-dependent (unlike for [dLPC]^−^) for both the saturated and desaturated PC isobars (see Fig. 2C, D). Curiously, these features were not symmetrical for either system. Both PC model systems contain a symmetric PC component (e.g., PC 16:0/16:0 and PC 18:2/18:2) and a nonsymmetric component (e.g., PC 14:0_18:0 and PC 16:0_20:4, respectively). The greatest deviations from linearity occurred when the nonsymmetric components were the minority species, but were over-represented based on FA product ions. In both model systems this deviation decreased as the sample was diluted, nearly approaching the ideal fit line representing a one-to-one correspondence. Above 50 mol%, the FA product ion response was sigmoidal and not responsive to dilution; however, even in the range of the highest deviance (80–95 mol% PC 16:0_20:4 or PC 14:0_18:0) deviance did not exceed 5 mol% from the predicted. Examination of the PC mixtures showed that, when using MS^3^ fragments, isobaric PCs can be determined at picomolar concentrations and FA product ions can be used to determine the composition of isobaric species.

### Determination of the *sn*-1 versus *sn*-2 positional isomers in PCs

**Fig. 3.**
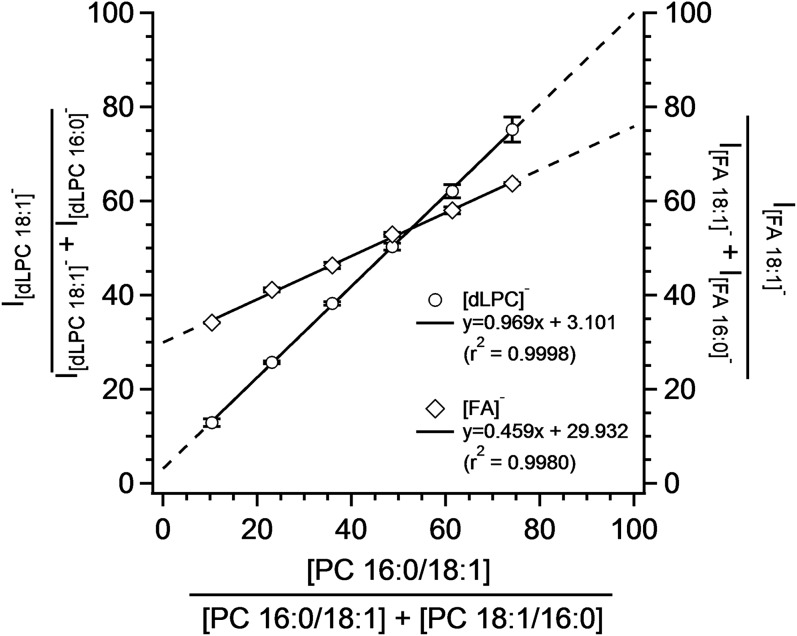
Dependence of the estimated mole percent of an isomeric PC mixture on the composition determined using [FA]^−^ and [dLPC]^−^ anions after MS^3^ fragmentation. Concentration of PC 34:1 was 0.065 nmol/ml. The MP consisted of a mixture of 5 mM ammonium chloride and ammonium acetate in a ratio of 1:4. Results are shown for acetate adducts. Bars represent mean ± SD of three repetitions.

**Fig. 4.**
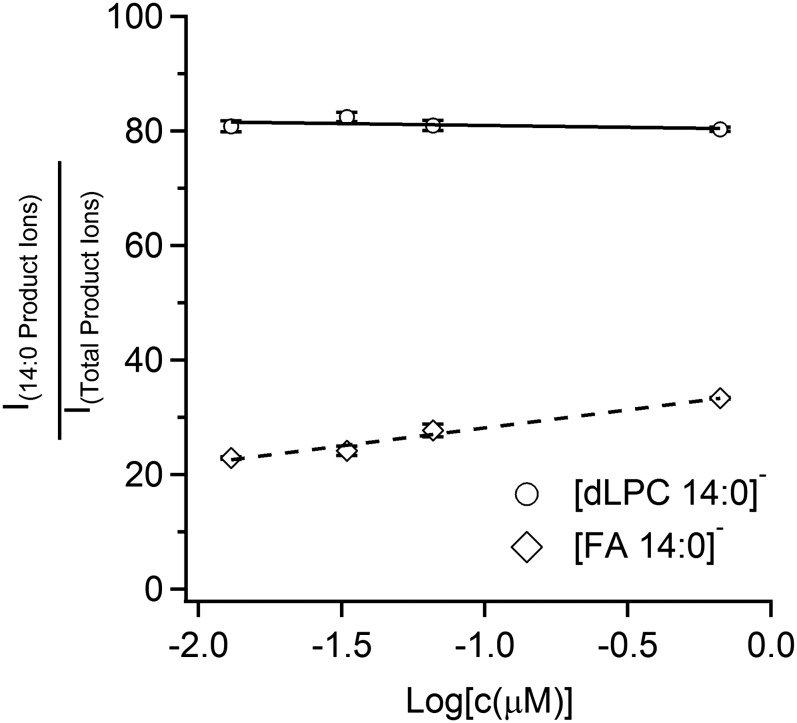
Concentration-dependence for determination of *sn*-1 versus *sn*-2 positional isomers. The graph shows a concentration influence on the signal ratios of anions [FA 14:0]^−^ and [FA 18:0]^−^, and [dLPC 14:0]^−^ and [dLPC 18:0]^−^ expressed as relative abundance (percent) of [FA 14:0]^−^ and [dLPC 14:0]^−^ in the MS^3^ spectrum of PC 14:0_18:0. PC was detected as acetate adduct in negative mode. The analysis was performed using a MP consisting of chloroform:methanol (1:1) with addition of 10 mM of ammonium acetate. Bars represent mean ± SD of three repetitions.

The isomeric purity of commercially available PC 16:0_18:1 and PC 18:1_16:0 was assayed by incubation with PLA_2_ and the resultant *sn1*_LPC species were quantitated using the PIS for *m/z* +184. Based upon these experiments, it was determined that the PC 16:0/18:1 standard contained 26% of PC 18:1/16:0 and the PC 18:1/16:0 standard contained 10% of the complementary PC 16:0/18:1 isomer. These findings comport with observations of previous researchers (25, 28). This information was used to determine the precise molar composition for six mixtures of these two isomers. Relative ratios of [FA]^−^ and [dLPC]^−^ product ions from the MS^3^ spectra versus the mole percent composition are shown in **Fig. 3**. While both sets of product ions display a relationship between ion intensity and mole ratio, the slope of the calibration curve was almost two times lower for FAs in comparison to the dLPC anions. In the case of dLPC fragments, the slope of the dependency was close to one. Further experiments showed that the ratio of the FA fragments slightly changes within the concentration of the isobaric PCs, while the ratio of dLPC fragments stays constant in the examined concentration range (see **Fig. 4**).

### Analysis of NIST human plasma

Using the methodology above, the SM, LPC, and PC species for NIST SRM for human plasma (SRM 1950) were quantified in a positive ESI PIS *m/z* +184 analysis (see supplemental Fig. S4). While plasmalogens, ether species, or PCs with odd chain FAs were observed, these results are not included in this work. Based on the PIS *m/z* +184 results, only PC signals with a relative intensity exceeding 1% of the base peak were targeted for negative MS^3^ scans. The selection contained 22 *m/z* signals. Below the concentration limit, the MS^3^ spectrum was not sufficient for proper quantitation, particularly of the positional isomers. Peak heights for both FA and dLPC anions were extracted from the MS^3^ spectrum and used to calculate the molar ratio of isobaric PCs in the mixture. Molar ratios from the MS^3^ experiments were applied to the positive mode results and isobaric species were expressed in nanomoles per milliliter.

A comparison of results using [FA]^−^ and [dLPC]^−^ fragments for the determination of isobaric PCs shows good agreement between both approaches (see **Table 1** and supplemental Table S1). Selective cleavage of the FA from the *sn-*2 position in the MS^3^ experiment allowed for determination of FA position on the glycerol backbone using [dLPC]^−^ fragments. In agreement with previous work, the saturated FA in the *sn-*1 position and the unsaturated FA in the *sn-*2 position considerably prevails (Table 1, supplemental Table S1) (28, 31). Furthermore, in PCs with two unsaturated FAs, the FA with the lower number of double bonds was preferentially in the *sn-*1 configuration.

**TABLE 1.** Analysis of an NIST human blood plasma SRM (SRM 1950)

						Isobaric PC Distribution				
PIS *m/z* +184		MS^3^		FA Moieties		[dLPC]^−^*^e^*,*^f^*		[FA]^−^*^f^*,*^g^*		
[PC+H]^+^ (*m/z*)	Brutto*^a^* Composition	[PC+59]^−^*^b^* (*m/z*)	[PC-15]^−^*^c^* (*m/z*)	*sn*-1*^d^*	*sn*-2*^d^*	Mean (nmol/ml)	SD (nmol/ml)	Mean (nmol/ml)	SD (nmol/ml)	Positional Isomer Ratio*^f^* (%)*^h^*
706.5	PC 30:0	764.5	690.5	C14:0	C16:0	2.1	0.1	2.1	0.1	—
730.5	PC 32:2	788.5	714.5	C14:0	C18:2	12.9	0.1	12.8	0.1	88
				C16:1	C16:1	1.7	0.0	1.8	0.0	—
732.6	PC 32:1	790.6	716.5	C14:0	C18:1	2.4	0.1	2.7	0.1	84
				C16:0	C16:1	13.2	0.3	12.8	0.3	83
734.6	PC 32:0	792.6	718.5	C16:0	C16:0	11.8	0.3	11.8	0.3	—
756.6	PC 34:3	814.6	740.5	C16:1	C18:2	7.7	0.1	8.5	0.1	87
				C16:0	C18:3	6.4	0.1	5.6	0.1	91
758.6	PC 34:2	816.6	742.5	C16:0	C18:2	403.1	4.7	403.1	4.7	89
760.6	PC 34:1	818.6	744.6	C16:0	C18:1	190.4	2.6	190.4	2.6	89
762.6	PC 34:0	820.6	746.6	C16:0	C18:0	1.9	0.3	1.9	0.3	—
780.6	PC 36:5	838.6	764.5	C16:1	C20:4	4.9	0.1	3.4	0.0	95
				C16:0	C20:5	8.7	0.1	10.1	0.1	94
782.6	PC 36:4	840.6	766.5	C16:0	C20:4	170.0	3.4	160.8	3.2	94
				C18:2	C18:2	16.1	0.3	25.3	0.5	—
784.6	PC 36:3	842.6	768.6	C16:0	C20:3	63.6	0.8	59.0	0.7	92
				C18:1	C18:2	68.5	0.9	73.1	0.9	86
786.6	PC 36:2	844.6	770.6	C18:0	C18:2	204.7	2.2	191.4	2.0	92
				C18:1	C18:1	21.7	0.2	32.1	0.3	—
				C16:0	C20:2	6.6	0.1	9.5	0.1	85
788.6	PC 36:1	846.6	772.6	C18:0	C18:1	32.7	0.6	32.0	0.6	88
				C16:0	C20:1	0.6	0.0	1.3	0.0	77
806.6	PC 38:6	864.6	790.5	C16:0	C22:6	42.6	0.8	40.3	0.8	95
				C18:2	C20:4	5.8	0.1	8.2	0.2	81
808.6	PC 38:5	866.6	792.6	C16:0	C22:5	19.6	0.2	18.8	0.2	94
				C18:1	C20:4	24.6	0.2	24.6	0.2	93
				C18:2	C20:3	2.1	0.0	2.7	0.0	66
				C18:0	C20:5	5.9	0.1	6.2	0.1	95
810.6	PC 38:4	868.6	794.6	C16:0	C22:4	7.1	0.2	8.6	0.3	91
				C18:1	C20:3	9.7	0.3	14.8	0.4	89
				C18:0	C20:4	106.1	3.1	99.6	2.9	94
812.6	PC 38:3	870.6	796.6	C18:0	C20:3	37.3	0.6	37.3	0.6	91
814.6	PC 38:2	872.6	798.6	C18:0	C20:2	9.3	0.3	9.8	0.3	86
				C18:1	C20:1	1.5	0.1	1.8	0.1	42
				C20:0	C18:2	2.5	0.1	1.7	0.1	85
832.6	PC 40:7	890.6	816.6	C18:1	C22:6	4.8	0.3	4.8	0.3	93
834.6	PC 40:6	892.6	818.6	C18:0	C22:6	16.9	0.6	16.6	0.6	95
				C18:1	C22:5	2.0	0.1	2.3	0.1	93
836.6	PC 40:5	894.6	820.6	C18:0	C22:5	9.6	0.2	9.4	0.2	93
				C18:1	C22:4	0.9	0.0	1.1	0.0	86
838.6	PC 40:4	896.6	822.6	C18:0	C22:4	4.4	0.2	4.4	0.2	91

a The numbers represent the total number of carbons: total number of double bonds in the acyl moieties in the PCs.

b First precursor, PC molecule with acetate adduct.

c Second precursor, demethylated PC molecule.

d Position of FA in the most abundant isomer (see Positional Isomer Ratio column).

e Determination of abundance of isobaric PCs using dLPC fragments.

f Results were obtained from four independent measurements.

g Determination of abundance of the isobaric PCs using FA fragments.

h Distribution of the isomeric PCs determined using dLPC fragments. The number indicates abundance of the PC having FA moieties in the configuration shown in the FA Moieties columns.

The influence of plasma matrix on isobaric and isomeric PC determination was evaluated by comparing results from crude plasma extract with those from plasma that had undergone SPE fractionation to isolate phospholipids from NLs (e.g., TGs and cholesterol esters). Analysis of the distribution of the isobaric PCs in the PL fraction resulted in an excellent agreement with the results found in the crude extract (supplemental Table S1).

### Comparison with other analytical techniques

**Fig. 5.**
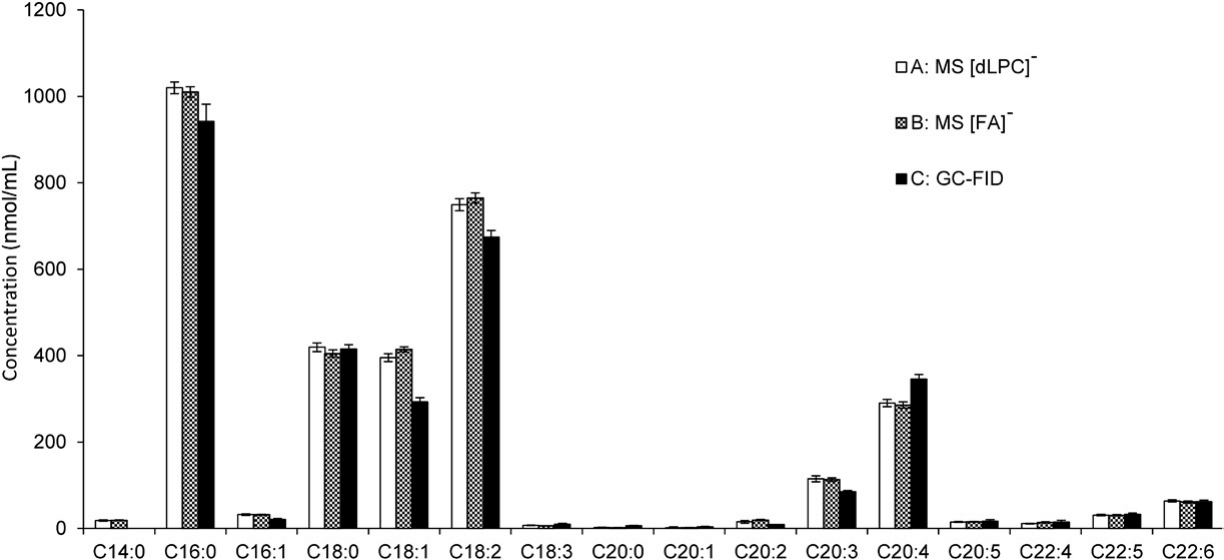
Comparison of FA distribution in the PL of a NIST human plasma sample obtained by two different analytical approaches. In the cases of A and B, samples were analyzed using PIS *m/z* +184 followed by MS^3^ fragmentation for isobaric PC determination. Isobaric PCs were determined using A [dLPC]^−^ and B [FA]^−^ anions. The obtained data was recalculated for the individual FAs. In the case of C, the FA distribution was determined using GC-FID as methyl esters after transesterification. Methyl tetradecanoate analyzed by GC-FID was not used due to the fact that the analysis was influenced by coelution with methylated BHT. Bars represent mean ± 99% confidence interval of four repetitions.

In order to support the quantitative nature of this shotgun approach, we compared the FA content of the PC calculated from the shotgun method to the traditional methods, including TLC and SPE followed by GC-FID. Because GC-FID requires transesterification treatment, all lipid species having FAs attached by an ester bond would contribute to the total signal of the resulting FA methyl esters. Therefore, we separated the crude extract of the NIST plasma sample by SPE into NL and PL fractions. The PL fraction was further analyzed by TLC and only spots corresponding to LPCs, SMs, and PCs were observed (see supplemental Fig. S5).This suggests that the abundance of other PL species, such as phosphatidylethanolamine, is very low, in agreement with previous studies performed on human plasma (1, 2, 40, 41). Then we analyzed PCs and LPCs in the PL fraction by MS using the newly developed approach. The remainder of the same sample was transesterified and analyzed by GC-FID. The quantified LPCs and PCs were recalculated into a contribution of the individual FAs using [FA]^−^, [FA-CO_2_]^−^, and [dLPC]^−^ MS^3^ fragments, and the results were compared with those obtained by GC-FID. The resulting total FA content from both methods was comparable (see **Fig. 5**).

## DISCUSSION

In this work, we present a targeted shotgun lipidomics approach for PC analysis that provides comprehensive structural and quantitative information. The novelty of this work is based on a combination of two scanning modes available on the same instrument, a hybrid triple quadrupole ion-trap mass spectrometer: *1*) PIS *m/z* +184; and *2*) MS^3^ scan in negative mode. The MS^3^ scan provides several types of fragments that contain both qualitative and quantitative information about PCs, including FA composition and position on the glycerol backbone. We examined two types of the resulting fragments, [FA]^−^ and [dLPC]^−^, and compared their analytical properties for isobaric and isomeric PC quantitation. The developed approach was then validated on a NIST human blood plasma SRM (SRM 1950) and the results compared with GC-FID (1).

Analysis of PCs is possible in both positive and negative modes. The detection of PCs as proton adducts in a positive mode (PIS *m/z* +184) is a highly sensitive method of quantitation due to the efficiency with which the choline head group is cleaved from the molecule; however, protonated PCs provide very little further structural information during fragmentation owing to the paucity of other fragments in positive mode (9, 35, 42, 43). While detection of PCs in the negative mode as acetate or chloride adducts is considerably less sensitive, multiple MS^n^ fragmentations in negative mode lead to analytically important ions useful for isobaric PC quantitation (44).

Based on earlier negative ESI MS^3^ studies, this current approach allows for improved selectivity toward PCs, as the MS^n^ fragmentation of the PC-anion adduct (forming the demethylated PCs) isolates PC species from the other isobaric phospholipids, including PCs with a different counter ion (28). As a consequence, the [FA]^−^ and [dLPC]^−^ fragments observed in the MS^3^ experiment are derived exclusively from isobaric PC species, with no interference from other isobaric species. Unlike previous studies, [FA]^−^ product ions, not [dLPC]^−^ product ions, were dominant features in the MS^3^ spectrum (see Fig. 1) (28). The relative intensity of these anions is not responsive to the optimization of parameters including excitation energy, and appears to be dependent upon the type of instrument used.

Our results show that both [dLPC]^−^ and [FA]^−^ fragments can be used for discriminating isobaric PCs into their various FA-containing constituents. A similar approach for the quantitation of isobaric species of phospholipids by employing [FA]^−^ fragments was recently reported (45). However, use of [FA]^−^ fragments only for isobaric PC determination may introduce error. We observed that the contribution of the [FA-CO_2_]^−^ product has to be considered for the quantitation. For example, in the case of PC 16:0_20:4, ion *m/z* 259 [FA 20:4-CO_2_]^−^ accounted for almost 20% of the product ions from fragmentation of polyunsaturated FA species in the MS^3^ spectrum (see supplemental Fig. S3). This observation is in agreement with other reports, although the relative abundance of the [FA-CO_2_]^−^ fragments in the MS^2^ or MS^3^ spectrum seems to be instrument dependent (11, 15, 46–48).

Differences in the intensities of [FA]^−^ and [dLPC]^−^ ions of a particular PC contain quantitative information about the representation of positional isomers. Measurements of isobaric PC mixtures showed that [dLPC]^−^ and [FA]^−^ product ions provide comparable quantitative results; however, only [dLPC]^−^ fragments provided a practically useful quantitation of positional isomers (see Fig. 3). The unsuitability of FA cleavage for positional isomer determination is discussed in numerous reports and is dependent on the nonselective nature of the generation of anionic product ions as well as the subsequent formation of [FA-CO_2_]^−^ (28, 46, 49). Thus, employing FA fragments for isomeric PC quantitation necessitates separate calibration curves for every PC containing FAs with three or more double bonds.

Characterization of the NIST human plasma sample served as one validation step for our method. This material was previously studied by the LIPID MAPS consortium; however, determination of isobaric and isomeric PCs is reported here for first time (1). Our results confirmed two general assumptions about PCs. First, the appearance of PCs with isobaric combinations of FAs is very common in the biological samples (15, 25, 28, 45). Second, saturated FAs occur preferentially in the *sn-*1 position (50). Furthermore, PC quantitation was not influenced by the presence of NLs such as TGs and cholesteryl esters (see supplemental Table S1). The results suggest that the analytical method is sufficiently robust as to be applied to the plasma crude extract without prior purification, which significantly simplifies sample preparation and reduces analysis time.

PCs in the NIST human plasma sample were previously analyzed on the level of brutto composition (see Table 1) by Quehenberger et al. (1). We observed several discrepancies between this previous report and our results (1) (see Table 1). Some of the most abundant PCs differ 2- to 3-fold in content (PC 34:2, PC 34:1, PC 36:1, PC 38:4, PC 38:2). Especially large differences were detected in a group of PCs with 40 carbons. PC 40:2 was reported in a concentration higher than100 nmol/ml, but we did not detect this PC (see Table 1) (1). Similar discrepancies were observed also by Holcapek et al. (2). However, our results, including LPCs (see below), are consistent with those reported by Holcapek et al. (2) and also other authors (42). It must be noted that these authors did not use the NIST standard. However, our analyses of SMs are in a good agreement with those obtained by Quehenberger et al. (1) (see supplemental Table S2). Differences in extraction procedures may be responsible for the discrepancy between Quehenberger et al. (1) and the other cited authors and this work (2, 42). Nevertheless, our data are supported by GC-FID analysis (see below).

Employing our method, the structure of SMs could not be determined for the lack of useful fragments in negative ESI MS^n^ (see supplemental Fig. S6). While not addressed in this work, the identification and quantitation of the most abundant LPCs can be performed directly from the PIS *m/z* +184 analysis (see supplemental Table S3). Analyses of ether-linked isobaric species of plasmanyl and plasmenyl analogs were not included in the study; nevertheless, preliminary results revealed that most of them are present in isobaric mixtures with PCs containing either odd chain or oxidized FAs (data not shown).

Quantitation of PC species using shotgun lipidomics provides an incredible depth of information with respect to molecular species structure. Supporting the quantitative nature of this shotgun approach is the very good agreement of FA content we obtained using the NIST plasma extract when compared with the more traditional method of PL separation followed by GC-FID analysis (Fig. 5). For some FAs, discrepancies between the MS and GC-FID methods were observed (e.g., C18:2 and C20:4). These differences likely result from the inability to correct for all extant influences of lipid structure upon ionization effects. Despite the observed deviations, the results indicate that quantitative shotgun lipidomics, in addition to being fast and internally consistent, can produce reliable results that parallel classical lipidomic methods.

Providing enhanced structural information for PC biomarkers may aid in the determination of disease mechanism or potential therapies. For example, Mapstone et al. (19) identified seven plasma PCs whose concentration changes indicate development of Alzheimer’s disease. However, the selected PCs were characterized only on the level of the brutto composition, the number of carbon and double bonds. PC 38:6 was among the listed biomarkers. Our analyses showed that PC 38:6 in human plasma consists of PC 16:0/22:6 (40.5 μM, 84%), PC 18:2/20:4 (4.7 μM, 10%), PC 22:6/16:0 (2.1 μM, 4%), and PC 20:4/18:2 (1.1 μM, 2%). From these data, we can speculate that n-3 and n-6 polyunsaturated FAs in the diet may influence the composition and concentration of this PC 38:6 biomarker.

In summary, we describe a method for providing quantitative data for the analysis of isobaric and regioisomeric PCs in human plasma. This method takes advantage the use of a hybrid quadrupole linear ion trap mass spectrometer and can be easily automated. Resulting information will allow more refined biomarker analysis in clinical studies.

## Supplementary Material

Supplemental Data
