# Supplementary material for: Quantitation of isobaric phosphatidylcholine species in human plasma using a hybrid quadrupole linear ion-trap mass spectrometer
Source: J Lipid Res. 2016 Nov 28;57(12):2225–34. doi: 10.1194/jlr.D070656 (PMC5321225; doi:10.1194/jlr.D070656)
Supplement: Supplemental Data [file supp_57_12_2225__index.html]

Quantitation of isobaric phosphatidylcholine species in human plasma using a hybrid quadrupole linear ion trap mass spectrometer — Quantitation of isobaric phosphatidylcholine species in human plasma using a hybrid quadrupole linear ion-trap mass spectrometer — Supplemental Data 

# Quantitation of isobaric phosphatidylcholine species in human plasma using a hybrid quadrupole linear ion-trap mass spectrometer

## Supplemental Data

- Supplemental Figs (.pdf, 384 KB) - Supplemental Figures
- MS3 isotopic correction (.pdf, 474 KB) - MS3 isotopic correction supplemental information
- Supplemental Tables (.xlsx, 35 KB) - Supplemental tables 1-3
