# Supplementary material for: Quantitation of isobaric phosphatidylcholine species in human plasma using a hybrid quadrupole linear ion-trap mass spectrometer
Source: J Lipid Res. 2016 Nov 28;57(12):2225–34. doi: 10.1194/jlr.D070656 (PMC5321225; doi:10.1194/jlr.D070656)
Supplement: Supplemental Data [file 10.1194_D070656_jlr.D070656-1.pdf]

SUPPLEMENTAL FIGURES:

Title: Quantitation of isobaric phosphatidylcholine species in human plasma using a hybrid quadrupole linear ion-trap mass spectrometer

Authors: Petr Žáček[1,2*], Michael Bukowski[1], Thad A. Rosenberger[3] and Matthew Picklo[1,4]

[1]USDA-ARS Grand Forks Human Nutrition Research Center; [2]Institute of Organic Chemistry and Biochemistry Academy of Sciences of the Czech Republic, Flemingovo nam. 2, 16610 Prague 6, Czech Republic; [3]Department of Biomedical Sciences, University of North Dakota School of Medicine and Health Sciences; [4] Department of Chemistry, University of North Dakota; Grand Forks, ND, 58201;

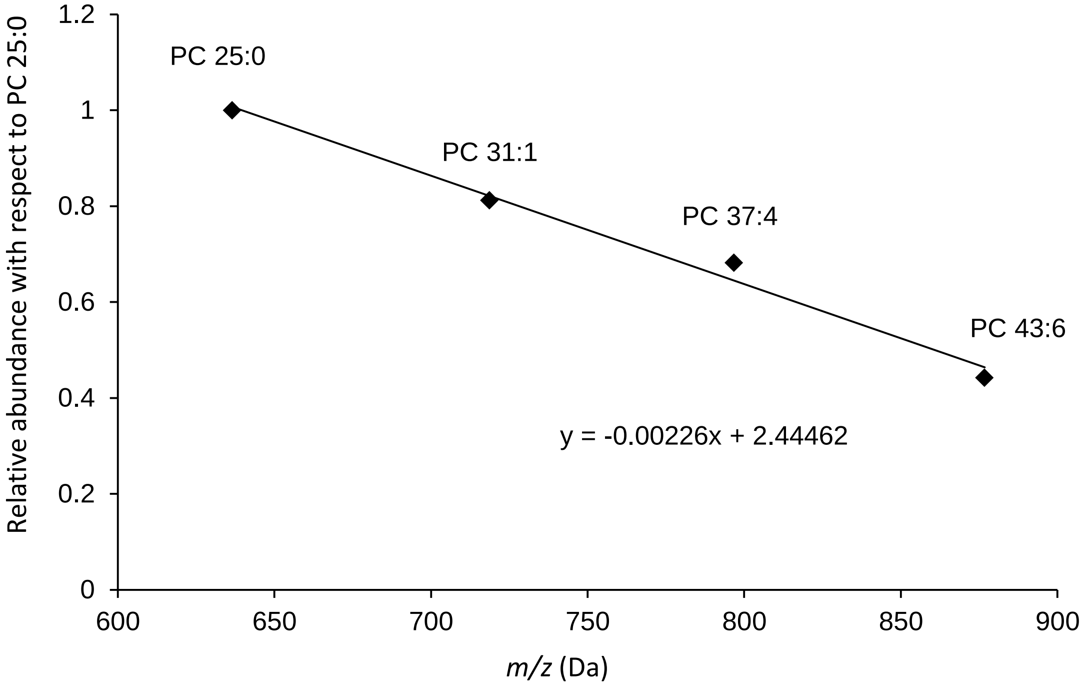

Supplemental Fig. S1: Dependence of the response of PC standards on number of carbons in the FA moieties. PC standards were present in the equimolar concentration and their signal was normalized with respect to PC 25:0. The equation of the linear model was used to correct of the ionization efficiency of the analyzed PCs on molecular mass. The slope of the dependency was used for correction of the ionization efficiency of SMs.

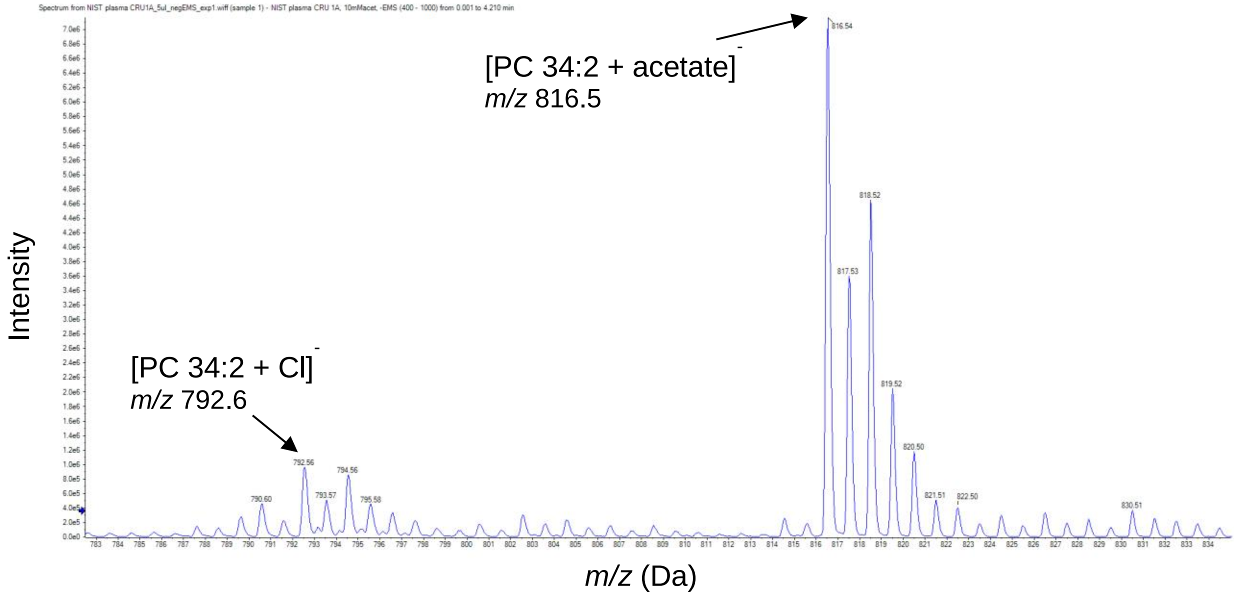

Supplemental Fig. S2: Mass spectrum of the mass range *m/z* 783 - 834 of crude extract of NIST human plasma extract in negative mode. The analysis was performed using mobile phase of chloroform:methanol (1:1) with addition of 10 mM of ammonium acetate.

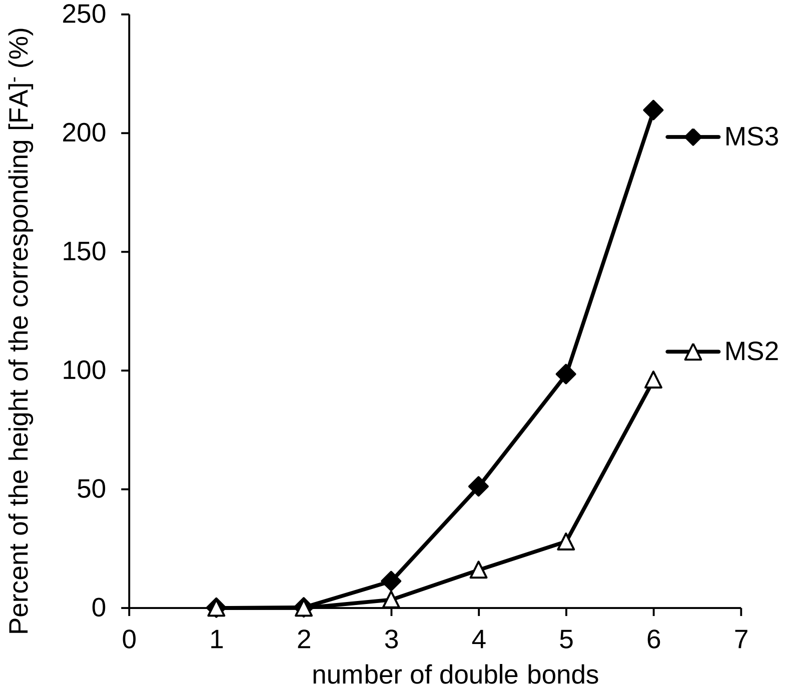

Supplemental Fig. S3: Dependence of the number of the double bonds in FA moiety and percentage ratio of the intensities between $[FA]^-$ signal after decarboxylation $[FA\text{-}CO_2]^-$ and the signal of the corresponding $[FA]^-$. PCs containing of the following FAs were used for the construction of the dependence: C18:1, C18:2, C18:3, C20:4, C22:5, C22:6. Position and configuration of the double bonds of the examined FAs was not determined. All PCs were detected as acetate adducts.

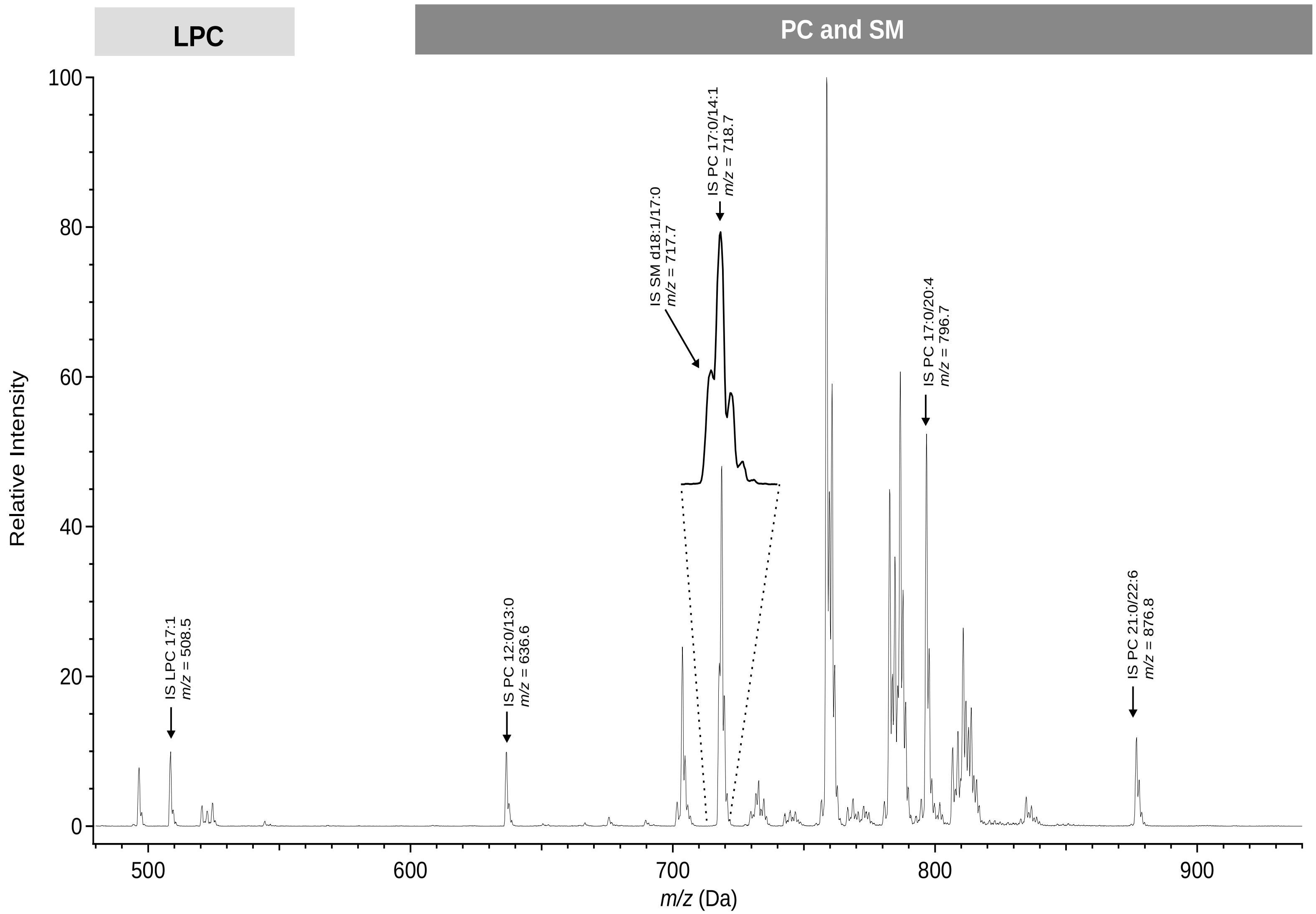

Supplemental Fig. S4: A PIS *m/z* +184 of the NIST human blood plasma standard with addition of the IS for quantification of the PCs, LPCs and SMs. The analysis was performed using mobile phase consisting of chloroform:methanol (1:1) with addition of 10 mM of ammonium acetate.

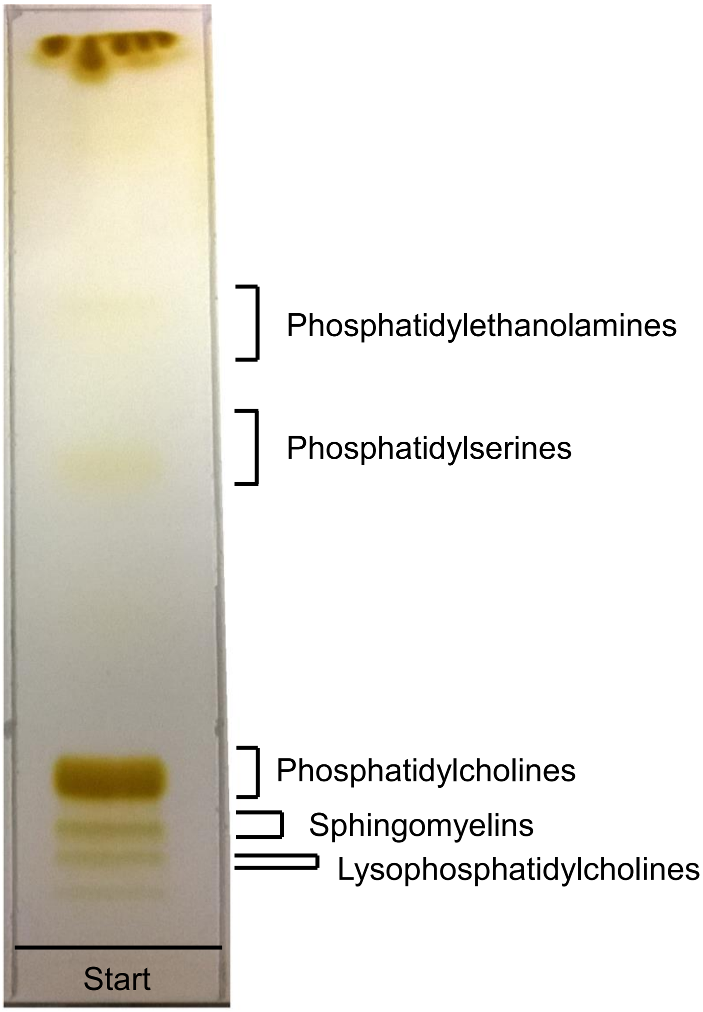

Supplemental Fig. S5: TLC of the crude extracts of the NIST human plasma. Mobile phase composition: $CHCl_3$: MeOH : AcCOOH : H_2O (50 : 37.5 : 3 : 2). Spots were visualized using iodine vapors. Phospholipid species were identified using standards.

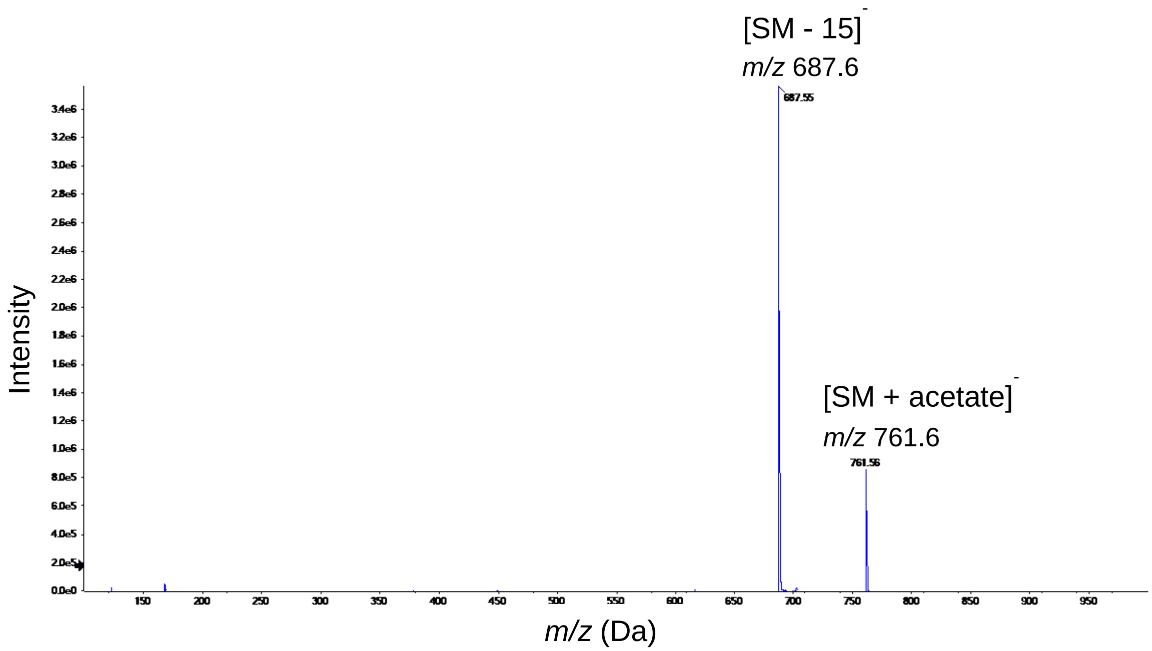

Supplemental Fig. S6: Averaged spectrum of SM d18:1_16:0 obtained after applying of collision energy range from -139 to -10 V on acetated adduct (*m/z* 761) in negative mode. Mobile phase used composed of chloroform:methanol 1:1 with 5 mM ammonium acetate. No ions corresponding to FAs moieties are present.
