# Supplementary material for: Quantitation of isobaric phosphatidylcholine species in human plasma using a hybrid quadrupole linear ion-trap mass spectrometer
Source: J Lipid Res. 2016 Nov 28;57(12):2225–34. doi: 10.1194/jlr.D070656 (PMC5321225; doi:10.1194/jlr.D070656)
Supplement: Supplemental Data [file 10.1194_D070656_jlr.D070656-2.pdf]

SUPPLEMENTAL INFORMATION:

Absolute quantitation of isobaric phosphatidylcholine species in human plasma using a hybrid quadrupole linear ion trap mass spectrometer

Petr Žáček[1,2], Michael Bukowski[1], Thad A. Rosenberger[3] and Matthew Picklo[1,4]

[1]USDA-ARS Grand Forks Human Nutrition Research Center; [2]Institute of Organic Chemistry and Biochemistry Academy of Sciences of the Czech Republic, Flemingovo nam. 2, 16610 Prague 6, Czech Republic; [3]Department of Biomedical Sciences, University of North Dakota School of Medicine and Health Sciences; [4] Department of Chemistry, University of North Dakota; Grand Forks, ND, 58201;

Isotopic correction of the MS^3 scan

Type 2 isotope corrections correct for the contribution of a lipid species containing two ^{13}C isotopes from the monoisotopic peak of a second species that has one greater point of saturation (1). In MS^2 and MS^3, the isotopically labeled “contaminant” species will produce fragments containing zero, one, or both ^{13}C atoms, M, M+1, and M+2, respectively. The monoisotopic “target” analyte will produce only monoisotopic fragments that will overlap with either the M or M+2 fragment. Calculating and matching the fragment distribution M+1 allows for prediction of the intensity and correction for the contaminating fragment. The relative intensities of M, M+1 and M+2 can be calculated as the product of two binomial coefficients. (eq. 1)

$$\binom{i}{x} \times \binom{N-i}{K-x} = \frac{i!}{x!(i-x)!} \times \frac{(N-i)!}{(K-x)!(N-i-K-x)!} = P_{M+x} \quad \text{(eq. 1)}$$

For a precursor ion of N carbon atoms containing *i* labeled ^{13}C atoms and producing product ions with K carbon atoms, the number combinations (P_{M+x}) that result in the incorporation of *x* ^{13}C atoms into the fragment (where x = 0, 1, 2 … *i*) can be calculated for each value of *x*. The result in the case where there are two ^{13}C atoms present in the molecule produces three values, P_M, P_{M+1}, P_{M+2}. Normalization to P_{M+1} provides relative ratios for these species.

The above model does not account for the isotopic contributions of ^2H or ^{18}O in FA and dLPC fragments which are smaller, and more static than the effect of changing carbon number for a PC species. While the same theoretical approach can be used to account for these contributions, an empirical approach is effective as well. Using a set of four standards (PC 16:0/16:0, PC 14:0_18:0, PC 18:2/18:2, and PC 16:0_20:4) empirical deviations from the model were determined for FA and dLPC fragments. The mean of these deviations was used to determine additive constant values to be included with the output for the normalized P_M, P_{M+1}, P_{M+2} values (Table 1).

Modeling the relative intensities to experimental data, it is possible to determine the portion of the experimental signal that belongs to M+2 contaminating species. The M+2 contribution of PC 16:0_18:1(blue) to PC 16:0_18:0 (red) is demonstrated in Figure 1. The intensity of the signal at *m/z* 283 is an admixture of FA 18:0 and $^{13}C_2$-labeled FA 18:1, with the former representing only about 20% of the signal. This is can be confirmed by looking at the additional intensity at *m/z* 255, where FA 16:0 is a common fragment for both species. In this example the

M+2 contribution is substantial because the concentration of the PC 34:1 species is 100-fold greater than that of the PC 34:0 species as measured by the precursor ion scan method (190 vs. 1.9 nmol/mL). This contamination arises in a handful of other few cases as well (PC 36:1, PC 38:3, and PC 38:2) however most cases this isotopic contribution is negligible.

1. Wang, M., C. Wang, and X. Han. 2016. Selection of internal standards for accurate quantification of complex lipid species in biological extracts by electrospray ionization Mass spectrometry-What, How and Why? *Mass Spectrom. Rev.* DOI 10.1002/mas.21492.

Table 1. Relative signal intensities for modeled PC 34:1 fragments.

Product ion	m/z	m/z	Model Signal Intensity (%)	Correction Constants (%)	Corrected Signal Intensity (%)
FA (18:1)	M	281.3	61	26	87
	M+1	282.3	100	0	100
	M+2	283.3	37	13	50
FA (16:0)	M	255.3	75	26	101
	M+1	256.3	100	0	100
	M+2	257.3	30	13	43
dLPC 16:0	M	480.3	37	5	42
	M+1	481.3	100	0	100
	M+2	482.3	61	31	92
dLPC 18:1	M	506.3	30	5	35
	M+1	507.3	100	0	100
	M+2	508.3	75	31	106

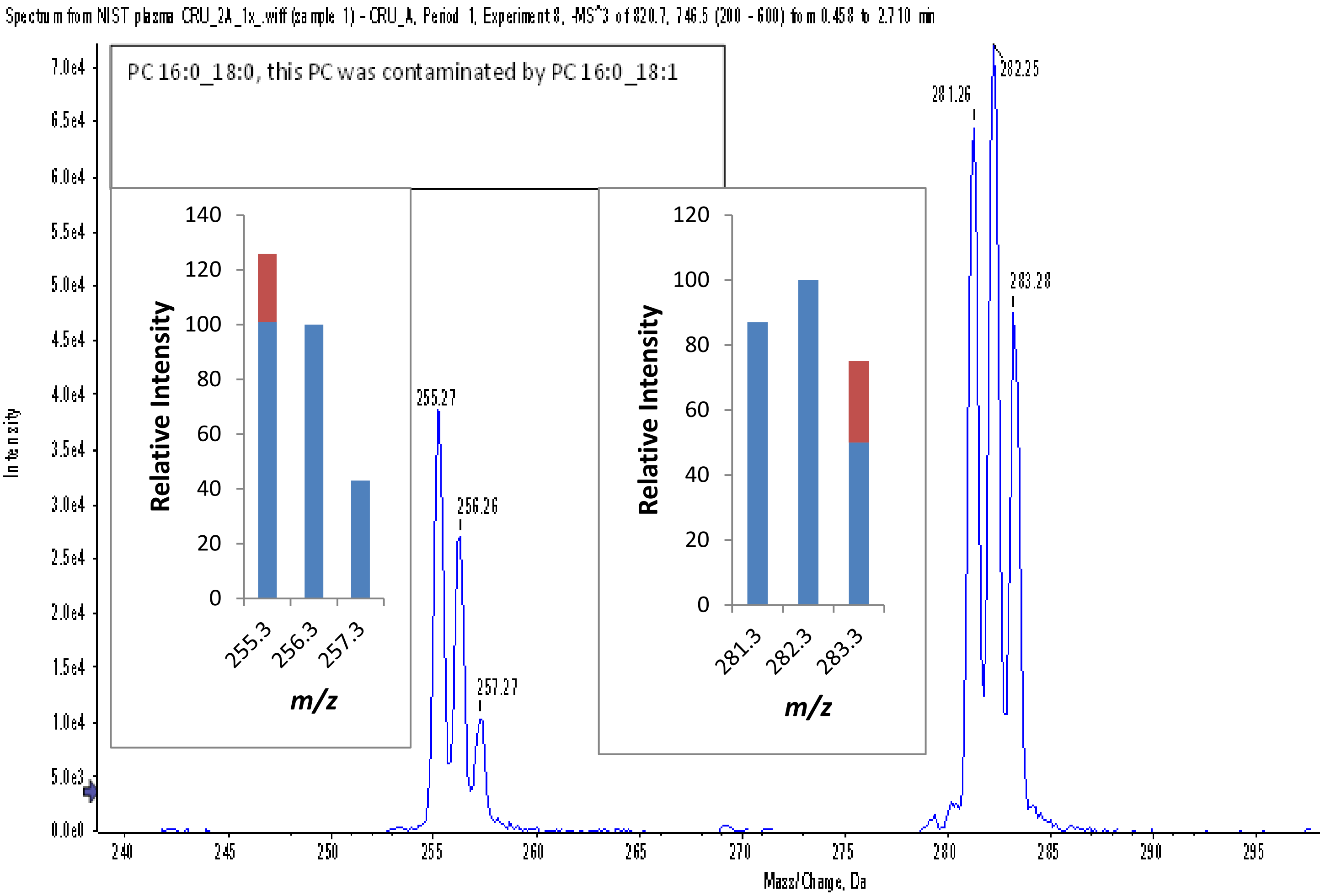

Figure 1. Comparison of experimental data to modeled relative intensity values (insets). The blue bars in the inset graphs represent modeled relative intensities of the particular FA isotopic peaks of PC 16:0_18:1. The red part of the bar represents the contribution of the contaminated PC 16:0_18:0.
